# Differential Plasma-cell evolution is linked with *Dermatophagoides pteronyssinus* immunotherapy response

**DOI:** 10.1038/srep14482

**Published:** 2015-09-29

**Authors:** Tahia D. Fernández, Enrique Gómez, Inmaculada Doña, Paloma Campo, Carmen Rondon, Miguel Gonzalez, Francisca Gomez, Francisca Palomares, Maria Salas, Miguel Blanca, Cristobalina Mayorga, Maria J. Torres

**Affiliations:** 1 Research Laboratory-Allergy Unit, IBIMA-Regional University Hospital of Malaga, UMA, Malaga, Spain; 2 Allergy Service, IBIMA-Regional University Hospital of Malaga, UMA, Malaga, Spain

## Abstract

Allergic rhinitis is highly prevalent worldwide. Immunotherapy has been shown to control its symptoms, however, up to 30% of patients may not respond. Previous studies of the immunological mechanisms involved in allergen-immunotherapy (AIT) have focused on the humoral and T-cell response and several studies have evaluated some B-cell subpopulations during AIT and their role in immunological tolerance. However, although B and plasma-cell subpopulations are two of the most important cellular subtypes involved in allergic reactions, their relation with AIT efficacy remains unelucidated. The objective was to analyze the effects of immunotherapy on different B and plasma-cell subpopulations and whether these changes correlate with the clinical response to the treatment. Although no changes are found in B-cell subpopulations, responder patients show increased levels of memory B-cells even before the beginning of treatment. Changes in plasma-cell subpopulations are found, mainly in circulating inflammatory plasma-cells that could affect the response to the allergen. Moreover, an early increase of specific-IgG4 and IgG4 secreting-cells was found. All these suggest that the determination of the memory B-cells before the initiation of the treatment, and the quantification of IgG4 and IgG4-secreting-cells in the first months of immunotherapy, could serve as markers for the clinical response to treatment.

In recent years the prevalence of allergic respiratory diseases has increased in western countries^1^; around 7% of the world´s population suffers from allergic rhinitis (AR)^2^. Management includes allergen avoidance, pharmacologic control of the symptoms and allergen-specific immunotherapy (AIT)^3^,^4^, the only etiologic treatment that affects the underlying immunopathological mechanism. AIT efficacy has been confirmed in systematic reviews and meta-analysis studies of asthma^5^,^6^ and more recently for AR^7^. Benefits are measured in terms of symptom reduction and improvements in quality of life^8^. Advantages of AIT over pharmacological treatment are: induction of disease remission over a long time^9^, prevention of new allergenic sensitizations^10^ and reduction of disease progression from AR to asthma^11^. Its efficacy has been demonstrated against highly prevalent allergens such as pollens and house dust mites^12^. However, up to 30% of patients do not respond to AIT^13^. More importantly, we cannot predict which patients will respond before beginning treatment, and since we are dealing with long-lasting treatments (up to five years) this implies a high cost to the health system especially for people that will not benefit from it.

Previous studies of the immunological mechanisms involved in AIT have focused on the humoral and T-cell response^14^, assuming that protection is associated with the induction of blocking antibodies. During AIT there are high levels of allergen-specific IgG1, IgG4 and IgA that can block the binding of the allergen-IgE complex at the surface of effector cells^15^,^16^. Specific IgG levels have been used as a biomarker to monitor AIT response^17^,^18^,^19^, although their utility for predicting treatment outcome has not been proven.

In the immunological mechanism underlying AR, B-cells produce specific IgE, antibodies that, due to their constant production by plasma cells, can be found in the serum for a long time^20^, sensitizing mast cells and basophils^21^. In the primary response, an activation process leads to the production of specific memory B-cells, responsible for long-term memory. Following subsequent contact with the allergen, memory B-cells differentiate into antibody secreting cell subpopulations^22^. Plasmablasts leave the lymph nodes and mature into plasma-cells. Some move to the bone marrow (long-lived), expressing the receptor CXCR4^23^,^24^,^25^ and can stay in the body for years^24^,^26^,^27^, or in the inflamed tissues (inflammatory plasma-cells)^28^, which express the migration-driving receptor CXCR3^23^,^24^,^25^. Inflammatory plasma-cells are responsible for increased antibody levels during an allergic response (Fig. 1).

Several studies have evaluated B-cell subpopulations during AIT and their role in immunological tolerance^29^,^30^. However, although B and plasma-cell subpopulations are two of the most important cellular subtypes involved in allergic reactions, their relation with AIT efficacy remains unelucidated.

Here, we analyse whether AIT can induce changes in B and plasma-cell subpopulations and if these changes correlate with clinical improvement. We have selected patients with AR, sensitized to the highly prevalent house dust mite *Dermatophagoides pteronyssinus* (DP) and examined differences in cell subpopulations between responders (RP) and non-responders (NRP) before and during treatment, attempting to find biomarkers for AIT effectiveness.

## Results

**Figure 2:**
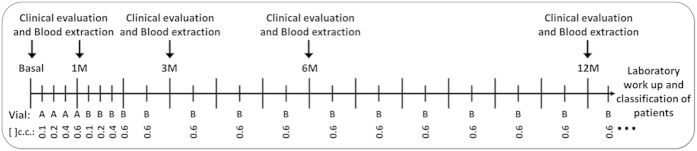
Experiment design. Time line representing the AIT schedule (bottom) and the different time points (basal, 1, 3, 6 and 12 months) in which patients were clinically evaluated and blood samples were taken. Blood samples were always obtained before AIT injection. All laboratory analysis were done in cryopreserved cells and frozen sera after the 12 months and before classification of the patients in RP and NRP.

Thirty-four patients (Table 1) were treated with subcutaneous AIT (Acaroid®, Allergopharma KG, Reinbek, Germany) for 12 months using a conventional schedule (Table S1 and Fig. 2), and none of them had adverse effects related to AIT. After 1 year, patients were classified into responder patients (RP, n = 28), based in their improvement >20% of the scores, and non-responder patients (NRP, n = 6) if they did not report improvements. Comparisons between RP, NRP and control group (CG, n = 14) showed that members of the NRP group had a longer duration of AR (180 months) compared to the RP (36 months) and CG (60 months; p = 0.0001) and were older than RP (p = 0.001) and CG (p = 0,030) (Table 1). There were no significant differences in sensitization to other allergens between groups.

In RP and NRP groups, AIT induced a tendency for a reduction in the size of the response to intradermal skin test and a slight increase in the allergen concentration required to induce a positive nasal provocation test. In CG we observed a slight increase in the intradermal test area and a significant decrease (p = 0.008) in the DP concentration inducing positivity in nasal provocation test (Table 1).

### AIT to DP induce changes in plasma cell subpopulations in responder patients during the first year of treatment

**Figure 3:**
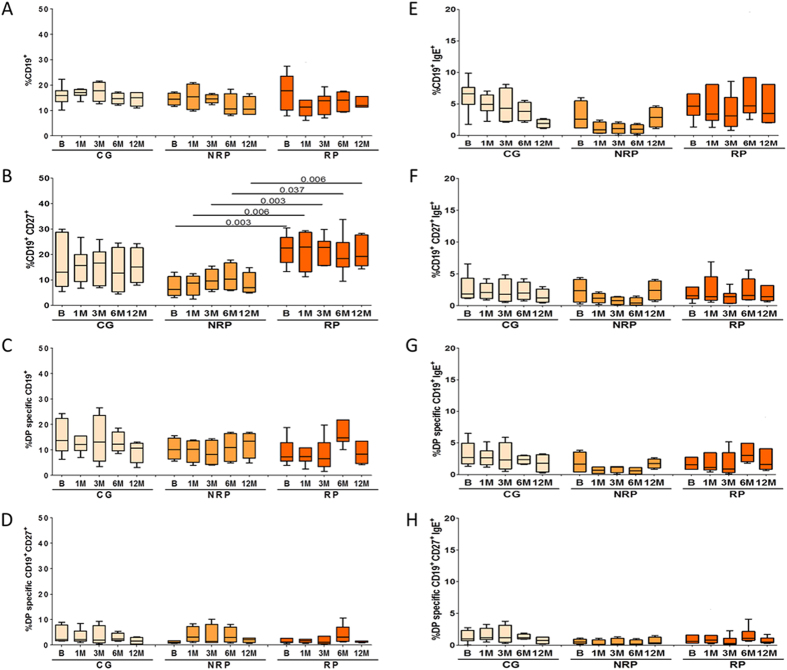
B-cell phenotyping. After 12 months of treatment, cryopreserved cells were stained to assess the evolution of different B cell subpopulations during AIT. Box plots represent the median and IQR of the percentage of cells of each phenotype. Data is shown for the CG, NRP and RP during 1-year follow-up of AIT (**A**) B-cells (CD19^+^); (**B**) Memory B-cells (CD27^+^); (**C**) Specific B-cells (DP^+^); (**D**) Memory Specific B-cells; (**E**) B-cells expressing IgE; (**F**) Memory B-cells expressing IgE; (**G**) Specific B-cells expressing IgE; (**H**) Memory Specific B-cells expressing IgE. Results for B cell subpopulations were expressed as percentage of CD19^+^ cells. Results for IgE expressing cells were expressed as percentage of each specific subpopulation. Statistical Wilcoxon and Mann-Whitney U tests were performed. The Bonferroni correction was applied for comparison of three groups and five different time points. No significant changes were observed for any group in any of the subpopulations analyzed. Only a significant higher percentage of memory B cells in RP at basal level were observed.

Using flow cytometry we evaluated changes induced by AIT in different B-cell subpopulations (total and specific B-cells, memory B-cells, B-regs, plasmablast and plasma-cells) in peripheral blood, as main effector cells, and their relation with treatment effectiveness. We found a significant difference in the percentage of circulating memory B-cells (CD27^+^) between RP and NRP (p = 0.003) at baseline that is maintained during the treatment (p = 0.006; p = 0.003; p = 0.037 and p = 0.006 at 1, 3, 6 and 12 months respectively), but without significant changes over AIT (Fig. 3B). Considering the surface expression of IgE by B-cell subpopulations, we did not observe any changes in any of the groups studied (Fig. 3E–H).

Regarding IL10-secreting-specific B-regs, no significant changes were found in any of the groups; however a tendency to increase was observed in RP, whereas no changes were found in NRP (Fig. S1). Interestingly a positive correlation between clinical response to treatment and IL10-secreting-specific B-regulatory cells was found from the first month of AIT and maintained throughout the treatment (p = 0.009; p = 0.027; p = 0.035 and p = 0.021 at 1, 3, 6 and 12 months respectively).

**Figure 4:**
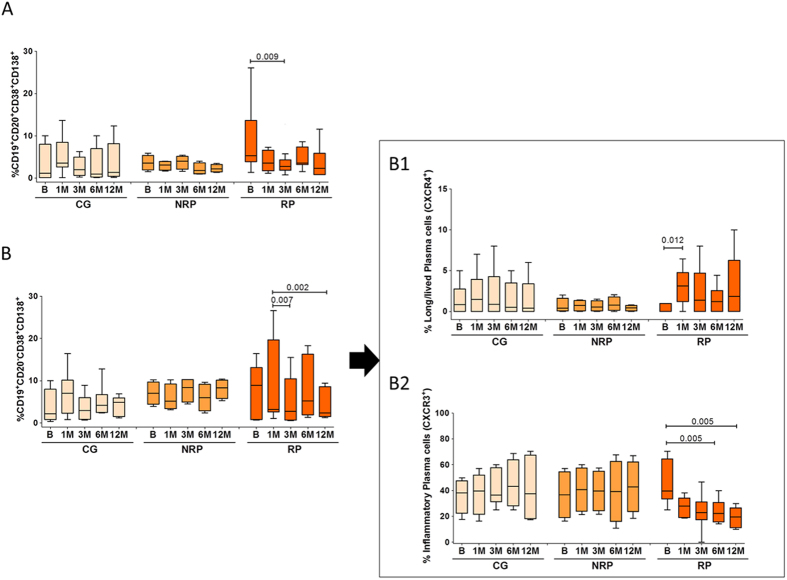
Plasma-cells phenotyping. Was done in cryopreserved cells after 1 year of AIT by flow cytometry. Box plots represent the median and IQR of the percentage of cells of each phenotype. Data is shown for CG, NRP and RP during 1-year follow-up of AIT. (**A**) Plasmablast (CD20^+^CD38^+^CD138^+^); (**B**) Plasma-cells (CD20^−^CD38^+^CD138^+^); Results for these cells were expressed as percentage of CD19^+^ cells. (B1) Long live Plasma-cells (CXCR4^+^); (B2) Inflammatory Plasma-cells (CXCR3^+^); these results were expressed as percentage of Plasma cells. A significant reduction of the percentage of inflammatory plasma-cells was observed during the AIT. Statistical Wilcoxon and Mann-Whitney U tests were performed. The Bonferroni correction was applied for comparison of three groups and five different time points.

**Figure 5:**
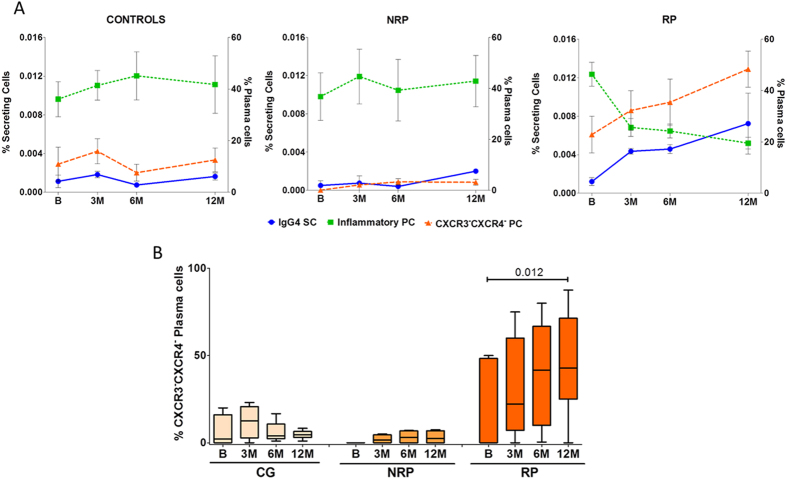
Co-evolution of Plasma-cells with IgG4 secreting cells in CG, RP and NRP. (**A**) Changes in percentage of IgG4 secreting cells over time determined by ELISpot showed an inverse evolution to the changes in the percentage of inflammatory plasma-cells (CXCR3^+^). On the contrary, the same evolution pattern was found in CXCR3^-^CXCR4^-^ plasma-cells in RP patients only. This result suggests the possibility they may be the same cells. (**B**) Box plots representing the median and IQRs of the percentages of CXCR3^-^CXCR4^-^ plasma-cells for each time point. An increase of these cells was observed in RP patients during AIT. Statistical Wilcoxon and Mann-Whitney U tests were performed and, applying Bonferroni correction, significant changes were considered when p < 0.012. Results for CXCR3^+^ and CXCR3^-^CXCR4^-^ cells were expressed as percentage of total plasma cells (CD19^+^CD20^-^CD38^+^CD138^+^).

Plasma-cells are known as the main source of IgE and thus considered as the effector cells in AR (Fig. 4). Plasmablast, plasma-cells precursors, showed a tendency to decrease significant at the third month of AIT (p = 0.009) (Fig. 4A). Total plasma-cells decreased after 3 months of AIT (p = 0.007 at 3 months; p = 0.002 at 12 months, both compared with the first month) (Fig. 4B). These plasma-cells can be classified as long-lived or inflammatory plasma-cells by their expression of CXCR4 or CXCR3 respectively. Data showed that the long-lived plasma-cells increased significantly at 1 month (p = 0.012), decreasing again in the following months (Fig. 4B1). Interestingly, circulating inflammatory plasma-cells showed a decrease from the first month, being significant at 6 and 12 months (p = 0.005 at 6 months; p = 0.005 at 12 months) (Fig. 4B2). We also found a tendency to increase in CCXR3^−^CXCR4^−^ plasma-cells during the treatment, being significant at 12 months (p = 0.012) (Fig. 5B). These changes were observed in RP only.

### AIT to DP induce a rapid decrease of sIgE/IgG4 ratio with an increase in IgG4 secreting cells in responder patients

**Figure 6:**
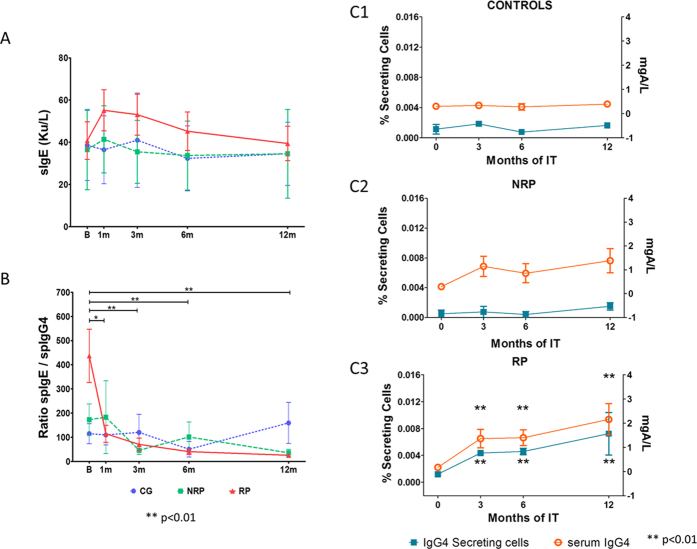
Evolution of Ig serum levels and IgG4 secreting cells in CG, RP and NRP. Ig levels in the different time points of the study were measured at the same time at the end of the first year of treatment. (**A**) Evolution of Total IgE (Ku/L). (**B**) Ratio between spIgE and spIgG4 in the three groups of patients represented as means and SD showing a significant decrease in RP from the first month of treatment. (**C**) Evolution of the percentage of IgG4 secreting cells determined by ELISpot and IgG4 serum levels determined by ImmunoCAP-FEIA in (C1) Controls, (C2) NRP and (C3) RP, where an increase of both parameters from the first month of AIT was observed. Statistical Wilcoxon and Mann-Whitney U tests were performed. The Bonferroni correction was applied for comparison of three groups and five different time points.

Total IgE, sIgE and sIgG4 were measured in sera collected at different time points during the treatment, to evaluate the effect of AIT on immunoglobulin production (Fig. 6). All the determinations were done at the same time and in a blind fashion. Total IgE and specific IgE and IgG4 were assessed by ImmunoCAP-FEIA. Serum DP-specific IgE showed a slightly, non-significant, increase in RP after 1 month and until 3 months, subsequently returning to basal levels (Fig. 6A). Analysis of the ratio between serum DP-specific-IgE and IgG4 at baseline showed a higher level in RP, although this was not significant. This ratio decreased significantly after the first month compared with basal, that was maintained throughout the first year of treatment (p = 0.011 at 1 month; p = 0.006 at 3 months and p = 0.0005 at both, 6 and 12 months) for RP only (Fig. 6B).

To assess if there was an increase in the cells able to secrete IgG4, we assessed the evolution of the percentage of IgG4 secreting cells by ELISpot assay at different time points during AIT. Data showed a significant increase in RP compared to CG at 3 months (p = 0.009, data not shown). Analysis of the data throughout AIT showed a significant increase for RP after 3 months compared to basal that was maintained until 12 months (p = 0.002 for all time points). The same pattern was found for serum specific IgG4 in RP (p = 0.005 at 3 months; p = 0.002 at 6 months and p = 0.0002 at 12 months) (Fig. 6C).

## Discussion

B-cells and plasma-cells are important targets for AIT immunomodulation in allergic diseases. There are few previous studies in this field, with some findings indicating that AIT can increase the percentage of memory B-cells^29^, specifically regulatory B-cells^29^,^30^. However, no study has been made of other cell subpopulations, e.g. plasma-cells, crucial for IgE production. Moreover, no previous work has compared changes in B-cell populations with AIT efficacy. In this study we have evaluated the changes in B and plasma-cell subpopulations in a group of AR patients sensitized to DP during the first year of AIT, comparing RP to NRP as well as non-treated patients. The aim was to identify the immunological changes associated with the clinical improvement. We observed that most changes were produced in RP at a very early time point in the AIT, with a decrease of inflammatory plasma-cells and an increase of cells secreting the blocking antibody IgG4, leading to a decrease in the IgE/IgG4 ratio.

Regarding clinical efficacy and based on symptom/treatment scores we expected to found a 30% of NRP as previously described^13^ that was account for approximately 10 patients, however we found that only 17.6% of cases did not respond to AIT. NRP patients showed a longer evolution time interval than RP and CG and, although not significant, were also older. Regarding objective measurements such as intradermal test and nasal provocation test, we found that while those receiving AIT showed a slightly smaller intradermal test area and no modifications in the nasal provocation test reactivity, non-treated CG responded to a lower concentration in the nasal provocation test and showed a slight increase in intradermal test area compared to baseline. These changes in intradermal test area agree with studies performed with grass AIT^31^, and could therefore indicate a worsening of the disease in those patients that do not receive AIT. Perhaps differences could also be found in RP and NRP over a longer time period; however this was not assessed here. Given that NRP did not show an increased nasal provocation test or decreased intradermal test, yet reported a worsening of symptoms (treatment/symptom scores, Table 1), we could speculate that NRP have chronic inflammation that could not be modified by AIT.

Surprisingly, B-cell subpopulations did not suffer any change directly induced by AIT, we only detected a tendency for an increase in DP specific memory B-cells in RP. These findings are different from those described by others in food IT^29^, where the authors detected an increase in memory B-cells after egg AIT, but the nature of the allergen and the exposition route was different and the patients were not continuously exposed. Moreover, we observed that the percentage of memory B cells was higher in RP compared to NRP before and during the treatment. As these cells are responsible for long term memory and the quick response in second contact with the allergen, their greater presence in RP could indicate a higher reactivity that could affect the treatment response. This could mean that there is a different immunological status between patients that are going to respond to the AIT (i.e. more reactive) compared to those that are not going to respond. If so, determination of memory B-cells before starting AIT could be used to predict future efficacy. Changes were less marked for regulatory B-cells than in previous studies performed with other allergens such as bee venom^30^,^32^,^33^, probably due to differences in the allergen exposure: intermittent, subcutaneous and at high doses in those studies, contrary to ours, in which exposure is continuous, intranasal and at low doses. However, there was a positive correlation between the percentage of IL-10 expressing regulatory B-cells and the clinical response, pointing to a role in the tolerance induction.

Plasmablasts, the intermediate stage between activated B-cells and plasma-cells^23^, and plasma cells can produce IgE^27^, however their evolution during AIT has not been studied. Moreover, although these cells are manly placed in secondary lymphoid tissues, it has been described their presence in peripheral blood of patients during an immune reaction^34^,^35^. Here we found a decrease in plasmablasts and circulating plasma-cells in RP, starting soon after AIT begins, which appears to be due to a decrease in circulating inflammatory plasma-cells, the cells recruited to the sites of inflammation^23^,^24^,^25^. An important future study would be to analyse the antibody isotypes produced by these cells, as they are likely to be the effector cells of the allergic episodes. In fact, decreases in this cell subset are found alongside a decrease in the serum specific IgE/IgG4 ratio. This decrease could be produced by the arresting of these cells in the tissues or by an overall reduction in the number of these cells. However, whatever the nature of this reduction in circulating inflammatory plasma-cells, it appears to correlate with successful treatment and could be useful as a clinical outcome biomarker. Regarding long-lived plasma-cells, which can stay in the body for years^24^,^26^,^27^ and that recirculate towards the bone marrow^23^,^24^,^25^, we found an increase after 1 month of treatment, however after this the levels returned to basal levels, which can be attributed to this recirculation.

Classically, the effect of AIT has been attributed to the increase of IgG4 in the humoral response^36^,^37^,^38^,^39^, since IgG4 can act as IgE-blocking antibodies^40^,^41^ disrupting other IgE-mechanisms like mast cell/basophil activation. We observed a significant and paralleled increase in serum specific IgG4 and IgG4 secreting cells in RP, and a significant decrease in the specific IgE/IgG4 ratio, which began at the start of AIT. The IgG4 secreting cells increase coincided with the decrease in inflammatory plasma-cells. Moreover, this increase matched with an increase in CXCR4^−^CXCR3^−^ plasma-cells, (Fig. 5) indicating that these could be responsible for the IgG4 secretion although further investigation is needed. However, there were no significant changes in the number of IgG4 secreting cells or the specific IgE/IgG4 ratio in NRP. We therefore speculate that both parameters could be useful as early biomarkers to determine which patients may respond to AIT.

One limitation of this study would be the low number of NRP that could underpower the statistical differences. However, this can be overtaken with the use of paired-tests, which compare related samples, in our case both, baseline versus 12 months. Moreover, this is the first study that investigates the plasma cell changes induced by AIT and its potential role as biomarkers of efficacy since main changes were observed in B-cell subpopulations from RP, in parallel with the Ig response.

In conclusion, our results revealed that successful AIT to DP was linked to a more reactive profile before starting treatment, with higher levels of memory B cells, and a substantial and maintained early decrease of this reactivity, with a reduction of inflammatory plasma-cells. This change could be responsible for reducing the ability of the immune system to quickly respond to the allergen and leads to a decrease in the immunological memory. This occurred in parallel with the decrease of IgE expressing B-cells, the specific IgE/IgG4 ratio and the increase in IgG4 secreting cells and serum IgG4 in the first year of treatment.

These results suggest a number of promising early biomarkers for determining whether a patient is likely to respond to AIT. This knowledge will help clinicians to monitor the patients during the treatment in order to make an early decision of whether or not to continue this long and expensive treatment potentially saving a great deal of time and money for the patient and the health system.

## Methods

### Patients work up

The study included 34 adult patients with an history of persistent AR to DP, diagnosed by skin prick test and specific IgE levels (ImmunoCAP-FEIA), treated with subcutaneous AIT (Acaroid®, Allergopharma KG, Reinbek, Germany) for 12 months using a conventional schedule (Table S1 and Fig. 2). All the cases were prospectively evaluated over a 1-year period and classified as RP and NRP based on the changes of total rhinoconjuntivitis symptom score (RCSS) and medication scores, following the indications of a recent EAACI (European Academy of Allergy and clinical immunology) position paper^42^, being defined as RP when both scores decrease by more than 20%. Patients were instructed to keep a diary during the previous month of all visits (baseline, 1, 3, 6 and 12 months), for a daily evaluation of the use of rescue medication and 5 rhinoconjunctivitis symptoms (sneezing, rhinorrhea, itchy nose, nasal obstruction, and ocular symptoms) according to a 4-point scoring system: 0, no symptoms; 1, mild symptoms; 2, moderate symptoms; and 3, severe symptoms. The maximum daily total symptom score was 15. The medication score was as follows: 1 point for oral antihistamines, 2 points if intranasal corticosteroids were also required, and 3 points if oral corticosteroids were also needed^42^. The change in the mean daily RCSS and medication scores obtained during the previous month of each visit were used to assess the clinical improvement in response to AIT. A control group (CG) (n = 14) of age- and sex-matched patients sensitized to DP with AR, with similar characteristics, but not receiving AIT, were included. The study was conducted according to the declaration of Helsinki and all patients participating in the study gave their informed consent and protocols were approved by institutional ethical committees (Comité Coordinador de Ética de la Investigación Biomédica de Andalucía).

### Nasal provocation test

Nasal provocation test was carried out as previously described^43^,^44^. Briefly, symptom-free patients (total VAS, <60 mm) were intranasally challenged with reconstituted freeze-dried allergen solutions of DP (0.04, 0.4 and 4 mg/mL) at 15-minute intervals. Two puffs (100 mL) of the solution at room temperature were applied in each nostril. Responses were monitored by means of acoustic rhinometry (SRE 2000 rhinometer, Rhinometrics, Lynge, Denmark) and symptom score. Acoustic rhinometry was performed following the guidelines of the Standardization Committee on Acoustic Rhinometry (E4), measuring the volume of the nasal cavity that corresponds to the lower turbinate (vol 2–6 cm) in each nostril. Symptoms of nasal obstruction, rhinorrhea, itching, sneezing and ocular symptoms were monitored at each time point by placing a vertical mark on a horizontal visual analogue scale (VAS) of 100 mm. The total range of the VAS during nasal provocation test was 0–500 mm. The response to nasal challenge was evaluated based on subjective (VAS of nasal-ocular symptoms) and objective (VOL 2–6 cm) parameters. A positive nasal provocation test response was considered to be an increase of 30% or greater in the total VAS score and a decrease of 30% or greater in the VOL 2–6 cm compared with the baseline test.

### Skin tests

Skin prick test was performed with a battery of 17 common inhalant allergens (ALK, Madrid, Spain). Intradermal skin testing was carried out with freshly reconstituted freeze dried DP (0.4 mg/mL) (ALK, Madrid, Spain) as previously described^45^, and results expressed as mm^2^.

### Sample collection and storing

Samples were obtained from subjects before starting AIT, and at 1, 3, 6 and 12 months during the procedure and immediately processed after their reception and frozen following current procedures (Fig. 2). These samples were managed and provided by the Málaga Hospital-IBIMA Biobank that also belongs to the Andalusian Public Health System Biobank. PBMCs, were obtained using isolation by Ficoll-Paque density gradient (GE Healthcare, Buckinghamshire, UK). Cells were stored in liquid nitrogen for further use. Serum samples were stored at −20 °C.

### Flow cytometry phenotype

Flow cytometry phenotyping and intracellular staining were carried out in cryopreserved cells after the 12 months of treatment and before classification as RP or NRP (Fig. 2) to preserved the blind, using specific fluorochrome-conjugated moAbs: CD19 PE-Cy7, CD19 APC-H7, CD24 PE, CD20 APC-H7, CXCR4 APC, CXCR3 PE (BD™ Bioscience, San Jose, CA, USA); CD27 PerCP-Cy5.5, CD38 AlexaFluor488, CD138 PerCP-Cy5.5 and IgE PE (Biolegend®, San Diego, CA, USA). DP1 was fluorescently labeled with AlexaFluor647 microscale protein labeling kit (Thermo Fisher Scientific, Massachusetts, USA). B-cell subpopulations were defined as (Fig. S2): CD19^+^ (B-cells); CD19^+^CD27^+^ (Memory B-cells); CD19^+^CD27^+^CD38^+^CD24^+^ (Breg); CD19^+^CD20^+^CD38^+^CD138^+^ (Plasmablast); CD19^+^CD20^−^CD38^+^CD138^+^ (Plasma-cells); once Plasma-cell subpopulation were difined the markers CXCR4 and CXCR3 were used to differentiate long-lived and inflammatory circulating plasma-cells respectively. Specific B-cells were selected by their ability to recognize fluorescently labeled Der p 1 (DP1), via their BCR. For intracellular staining, cells were fixed with BD Cytofix/CytoPerm Fixation/Permeabilization solution kit (BD™ Bioscience) and specific PE Cy7-conjugated moAbs to IL10 (eBioscience, San Diego, CA, USA) were used to determine its expression in Bregs. Dead cells were excluded using Far Red fluorescent or Near-IR fluorescent LIVE/DEAD® Fixable Dead Cell dyes (Thermo Fisher Scientific, Massachusetts, USA) depending on the antibody panel. Matching isotype controls were used as negative controls. Cells were acquired in a BD™ FACSCanto II flow cytometer and analyzed with FlowJo® software (Tree Star, Inc. USA). Results were expressed as % of total lymphocytes for B cells and percentage of CD19^+^ cells for the different subpopulutaions of B cells. For Plasma cells, results were expressed as percentage of CD19^+^CD20^+^ cells for plasmablast, percentage of CD19^+^CD20^−^ for plasma cells and percentage of plasma cells for the different subpopulations.

### ELISpot Assay

The number of IgG4 secreting cells was measured by a modified ELISpot assay originally described by *Czerkinsky et al.*^46^. A total of 2×10^5^ cells were seeded and incubated for 12 days at 37 °C and a 5% of CO_2_. This long time culturing has demonstrated to be useful for evaluating antibody secreting cells^47^. To measure the total number of Ig-secreting cells, samples were titrated (1:2 dilutions). Alkaline fosfatase labeled anti-IgG4 antibodies (Southern Biotech, Birmingham, AL, USA) were used overnight at 4 °C for detection. The colorimetric reaction with a ready to use solution of NBT-BCIP (Sigma-Aldrich, St. Louis, MO, USA) was allowed to proceed until spots were clearly evident. The reagent and plate backing material were removed, and the reactions were quenched by being submerged in distilled water. The number of IgG4 secreting cells was determined by counting the formed spots using a magnifying glass. Results were expressed as percentage of IgG4 secreting cells calculated as the ratio IgG4 secreting cells/total seeded cells.

### Total and specific IgE, IgG4 assays

Total and specific IgE and IgG4 to DP were determined by ImmunoCAP-FEIA in serum samples, according to manufacturer instructions (Thermo Fisher Scientific, Massachusetts, USA).

### Statistical Analysis

Quantitative variables were analyzed using the non-related Mann-Whitney U test, Kruskal-Wallis test and X^2^-test. Comparisons of related samples were carried out by Wilcoxon test. Correlation analysis was performed by Spearman Test. All reported p-values represented two-tailed tests, with values p ≤ 0.05 considered statistically significant. The Bonferroni correction was applied for multiple comparisons testing.

## Additional Information

**How to cite this article**: Fernández, T. D. *et al.* Differential Plasma-cell evolution is linked with *Dermatophagoides pteronyssinus* immunotherapy response. *Sci. Rep.* **5**, 14482; doi: 10.1038/srep14482 (2015).

## Supplementary Material

Supplementary Information

## Figures and Tables

**Figure 1:**
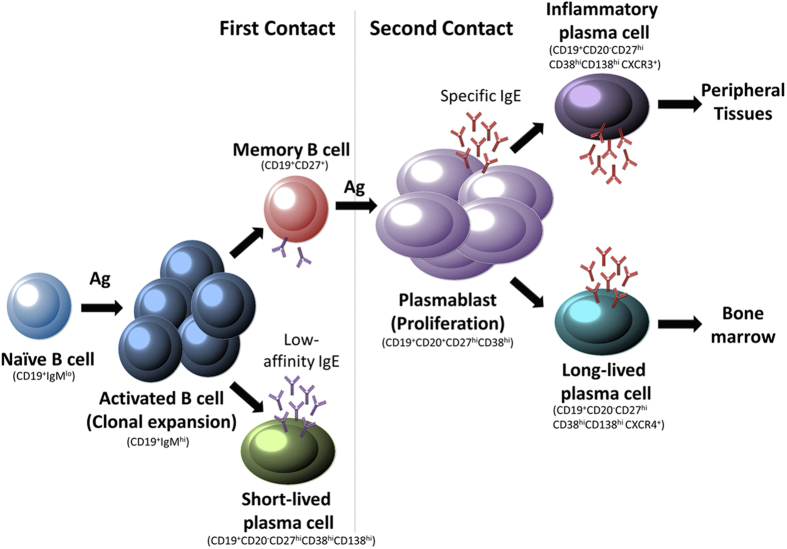
Proposed model representing the B cell subtypes involved in the development of the AR. In first contact, the allergen is presented to naïve B-cells; these activate and begin somatic hypermutation and class-switch recombination. Some of them become short-lived plasma-cells able to secrete low-affinity IgE as the first step of immunological protection. Another subset of activated B-cells becomes Memory B-cells. In successive contact with the allergen the memory B-cells differentiate into plasmablast that are able to secrete spIgE and proliferate, differentiating into: Long-lived plasma-cells, that preferentially recirculate to Bone Marrow, and Inflammatory plasma-cells, that are recruited to the peripheral tissues and act as the real effector cells with the secretion of spIgE. This proposed model is based on our current knowledge of IgG responses.

**Table 1:** Clinical characteristics for the three groups of patients included in the study.

	Sex	Age	Evolutiontime(months)	Otherallergens	RCSS			Medication score			Intradermal test to DP (mm2)			Concentration DP at positivenasal provocation test		
					Basal	12 month	p	Basal	12 month	p	Basal	12 month	p	Basal	12 month	p
RPn = 28	35.7%Females	28(21–31)	36(24–120)	83.3%	2,17(1,35–2,92)	1,55(1,32–2,85)	0.002	0,54(0,07–1,2)	0,25(0,07–1,21)	0.050	149(90–168)	100(88,5–148)	0.084	0,04(0,04–4)	0,04(0,04–0,4)	0.473
NRPn = 6	66.7%Females	33(30–33)	180(180–240)	66.6%	3,85(1,58–4,95)	8,32(6,52–9,43)	0.063	0,25(0,12–0,42)	0,67(0,57–0,88)	0.458	172(168–176)	109,5(99–120)	0.157	0,04(0,04–0,4)	0,04(0,04–0,04)	0.157
CGn = 10	40%Females	19(19–31)	60(24–120)	60%	4,745(1,46–5,89)	5,53(0,92–7,6)	0.122	0,07(0–0,41)	0,46(0–2,03)	0.458	132(118,25–189)	143(83,5–191,25)	0.916	0,4(0,04–0,4)	0,04(0,04–0,04)	0.008

RCSS: Total rhinoconjuntivitis symptom score; DP: Dermatophagoides pteronyssinus; RP: responder patients; NRP: Non responder patients; CG: Control group.

Data are represented as medians and range. Statistical analysis for two related samples were carried out by Wilcoxon test, values p ≤ 0.05 considered statistically significant.
