# Supplementary material for: Differential Plasma-cell evolution is linked with Dermatophagoides pteronyssinus immunotherapy response
Source: Sci Rep. 2015 Sep 29;5:14482. doi: 10.1038/srep14482 (PMC4586444; doi:10.1038/srep14482)
Supplement: Supplementary Information [file srep14482-s1.pdf]

# **Title:** **Differential Plasma-cell evolution is linked with *Dermatophagoides pteronyssinus* immunotherapy response.**

**Authors**:

Tahia D Fernández [1†], PhD; Enrique Gómez[1†], PhD; Inmaculada Doña[2], MD, PhD; Paloma Campo[2], MD, PhD; Carmen Rondon[2], MD, PhD; Miguel Gonzalez[1], BS; Francisca Gomez[2], MD, PhD; Francisca Palomares[1], PhD; Maria Salas[2], MD, PhD; Miguel Blanca[2], MD, PhD; Cristobalina Mayorga[1,2*], PhD and Maria J Torres[2*], MD, PhD.

[1]Research Laboratory-Allergy Unit, IBIMA-Regional University Hospital of Malaga, UMA, Malaga, Spain.

[2]Allergy Service, IBIMA-Regional University Hospital of Malaga, UMA, Malaga, Spain.

[†] Both authors contributed equally in this manuscript

[*] Both authors contributed equally in this manuscript

**Corresponding author and Address for reprint requests:**

Maria Jose Torres

Allergy Service

Pavilion 5, basement,

University Hospital of Malaga (Pavilion C).

Plaza del Hospital Civil. 29009 Malaga, Spain

Tel: +34 951290224. FAX: +34 951290302.

E-mail: mjtorresj@ibima.eu

## Supplemental Figures

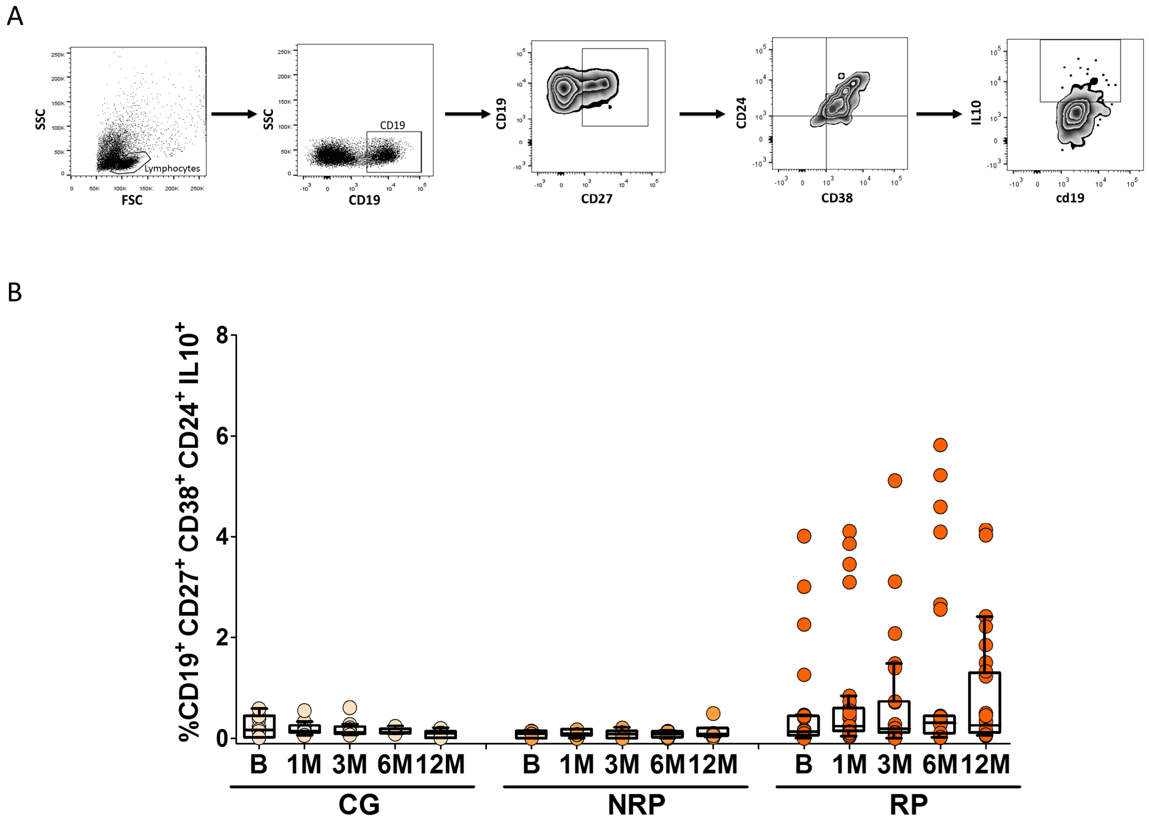


**Figure S1**. **Evolution of IL10 secreting Bregs in CG, RP and NRP during 1-year of AIT.** A) Flow Cytometry strategy followed to define Bregs and IL10 secreting Bregs. B) Individual data points and box plots representing the median and IQR of the percentage of $IL10^{+}$ Breg for each time point. We can observe a tendency suggesting a potential increase in RP patients during AIT, although this was not significant.

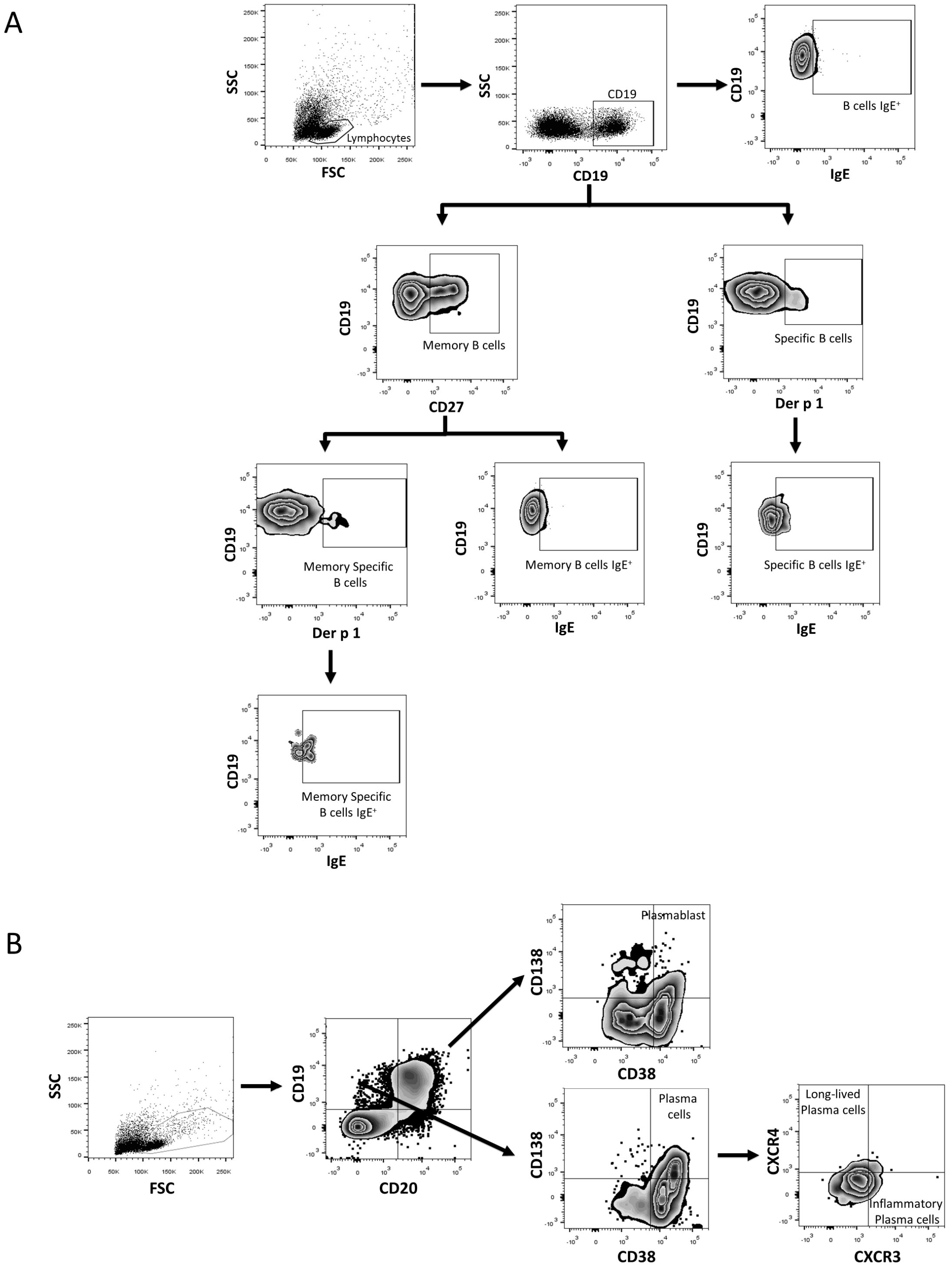
A
SSC
FSC
Lymphocytes
CD19
B cells IgE+
IgE
Memory B cells
CD27
Specific B cells
Der p 1
Memory Specific
B cells
Memory B cells IgE+
Specific B cells IgE+
Memory Specific
B cells IgE+
B
CD20
CD138
CD38
Plasmablast
Plasma
cells
Long-lived
Plasma cells
Inflammatory
Plasma cells
CXCR4
CXCR3

**Figure S2**. **Flow cytometry gating strategies for the different subpopulations analyzed.** A) Strategy to define the different B-cell subpopulations. Results were expressed as percentage of lymphocytes for B-cells, and as percentage of $CD19^+$ cells for the other subpopulations. B) Strategy to select plasma-cell subpopulations. Results of plasmablast and plasma-cells were expressed as percentage of lymphocytes; for long-lived and inflammatory plasma-cells, results were expressed as percentage of total plasma-cells. Positive and negative gates were determined using isotypic controls for each antibody.

**Table S1. AIT Schedule**

| Vial | Interval time of dose administration | Doses administered (cc) |
|---|---|---|
| **A** | 7 days | 0.1 |
| | | 0.2 |
| | | 0.4 |
| | | 0.6 |
| **B** | 7 days | 0.1 |
| | | 0.2 |
| | | 0.4 |
| | | 0.6 |
| | 14 days | 0.6 |
| | 1 month | 0.6 |
